# Therapeutic Efficacy and Safety of Osteoinductive Factors and Cellular Therapies for Long Bone Fractures and Non-Unions: A Meta-Analysis and Systematic Review

**DOI:** 10.3390/jcm11133901

**Published:** 2022-07-04

**Authors:** Angelos Kaspiris, Argyris C. Hadjimichael, Elias S. Vasiliadis, Dionysios J. Papachristou, Peter V. Giannoudis, Elias C. Panagiotopoulos

**Affiliations:** 1 Laboratory of Molecular Pharmacology, Department of Pharmacy, School of Health Sciences, University of Patras, 26504 Patras, Greece; 2 Department of Orthopaedics, St. Mary’s Hospital, Imperial College Healthcare NHS Trust, Praed Street, London W2 1NY, UK; ortho.argiris@gmail.com; 3 Third Department of Orthopaedic Surgery, School of Medicine, “KAT” General Hospital, National and Kapodistrian University of Athens, 2 Nikis Street, 14561 Athens, Greece; eliasvasiliadis@yahoo.gr; 4 Laboratory of Bone and Soft Tissue Studies, Department of Anatomy-Histology-Embryology, University Patras Medical School, 26504 Patras, Greece; papachristoudj@med.upatras.gr; 5 Academic Department of Trauma and Orthopaedics, School of Medicine, University of Leeds, Leeds LS7 4SA, UK; pgiannoudi@aol.com; 6 NIHR Leeds Biomedical Research Centre, Chapel Allerton Hospital, Leeds LS7 4SA, UK; 7 Department of Trauma and Orthopaedics, Patras University Hospital and Medical School, 26504 Patras, Greece; ecpanagi@med.upatras.gr

**Keywords:** open and closed long bone fractures, non-union, osteoinduction, BMPs, PRPs, MSCs

## Abstract

Background: Long bone fractures display significant non-union rates, but the exact biological mechanisms implicated in this devastating complication remain unclear. The combination of osteogenetic and angiogenetic factors at the fracture site is an essential prerequisite for successful bone regeneration. The aim of this study is to investigate the results of the clinical implantation of growth factors for intraoperative enhancement of osteogenesis for the treatment of long bone fractures and non-unions. Methods: A systematic literature review search was performed according to the Preferred Reporting Items for Systematic Reviews and Meta-Analyses (PRISMA) guidelines in the PubMed and Web of Science databases from the date of inception of each database through to 10 January 2022. Specific inclusion and exclusion criteria were applied in order to identify relevant studies reporting on the treatment of upper and lower limb long bone non-unions treated with osteoinductive or cellular factors. Results: Overall, 18 studies met the inclusion criteria and examined the effectiveness of the application of Bone Morphogenetic Proteins-2 and -7 (BMPs), platelet rich plasma (PRP) and mesenchymal stem cells (MSCs). Despite the existence of limitations in the studies analysed (containing mixed groups of open and close fractures, different types of fractures, variability of treatment protocols, different selection criteria and follow-up periods amongst others), their overall effectiveness was found significantly increased in patients who received them compared with the controls (I^2^ = 60%, 95% CI = 1.59 [0.99–2.54], Z =1.93, *p* = 0.05). Conclusion: Administration of BMP-2 and -7, PRP and MSCs were considered effective and safe methods in fracture treatment, increasing bone consolidation, reducing time to repair and being linked to satisfactory postoperative functional scores.

## 1. Introduction

Long-bone fractures, including femoral, tibial and humeral, represent one of the most frequent types of non-fatal traumas worldwide [1]. Although criteria for conducting the epidemiological surveys about musculoskeletal injuries vary from study to study, the overall prevalence of long-bone fractures was estimated at 406 per 100,000 people per annum [2], being higher for the adult population [3]. The frequency of humeral fractures in particular, which account for 0.5 to 3% of all fractures [4], is increasing with population aging, with significant socioeconomical effects [5].

Overall post-operative outcomes and complication rates are not well established for these injuries and depend on anatomic location, the severity of the accompanying soft-tissue injury, the patients’ comorbidities and the fracture fixation technique [6]. Prospectively collected UK national data demonstrated that the rate of non-union after long bone fracture treatment was between 1.9 and 10%, and depended on the type of fracture and the age group [6,7]. The same study group also analysed the fractures of long bones that were treated between 2005 and 2010 in Scotland; it reported that the overall prevalence of non-union was 18.94 per 100,000 population per annum [8]. Similarly, in the USA, the annual incidence of fracture non-union was estimated at 100,000 cases [6,9]. Regarding the association between anatomical fracture location and compromised bone healing, it was reported that the overall non-union rate of tibial, femoral and humeral shaft fractures after intramedullary nailing was 4.6% [6,10], 8% [6,11] and 33% [4,6] respectively. Moreover, analysis of 1106 cases of tibial fractures treated with reamed intramedullary nailing showed that the non-union rate of open fractures for Gustilo–Anderson type I–II and type IIIB was 42.1% and 69.2%, respectively [6,12].

Strategies to enhance bone repair during fracture non-union treatment include autologous or synthetic bone and allogeneic or xenograft grafting, implantation of growth factors, progenitor cells and/or combination of graft materials. Fixation techniques that apply in the treatment of long bone fractures include intramedullary nailing (IMN), open reduction and internal fixation (ORIF) and external fixation. Ilizarov external fixation, used for the treatment of large bone defects, pseudarthrosis, limb deformities and lengthening, is a very popular intervention, and it is associated with early weight bearing and increased rates of beneficial functional outcomes. Moreover, it allows minor post-operative corrections of the limb axis. The effectiveness of the Ilizarov technique in the treatment of comminuted tibial fractures with large bone defects was confirmed by a recent study that reported beneficial clinical outcome scores for lower extremity function and relatively low rates of post-operative infections [13].

Current progress in the research of the molecular pathways involved in the osteoinductive and angiogenic processes of bone regeneration encouraged the clinical application of growth factors and cellular therapies in the treatment of non-union and comminuted fractures. Although many studies reported that the application of these factors was correlated with increased bone healing rates [6,14,15,16,17], some others described low osteoinductive activity and a significant rate of complications such as post-operative infections [6,14]. Therefore, further research is needed to clarify the clinical potency of these therapies on patient recovery. The aim of this study is to compare the safety and effectiveness of the use of osteoinductive factors and cellular therapies for the treatment of close and open long bone fractures and non-unions.

## 2. Materials and Methods

### 2.1. Research Strategy

A systematic computer-based literature review search with predefined criteria was performed from the date of inception of each database up to 10 January 2022 according to the Preferred Reporting Items for Systematic Reviews and Meta-Analyses (PRISMA) guidelines [18] in the following repositories: PubMed (1947 to present) and Web of Science (1900 to present). The research methodology used a combination of the following terms: “long bone fractures [All Fields]”, “Osteoinduction [All Fields]”, “Bone Morphogenetic Proteins, BMPs [All Fields]”, “Femur [All fields]”, “Tibia [All fields]”, “Humerus [All fields]”, “Platelet Rich Plasma, PRP [All Fields]” and “Mesenchymal stem cells, MSCs [All Fields]”. The electronic literature search was conducted independently by two authors (E.P., A.K.) and an experienced librarian. Moreover, the above two authors (E.P, A.K) independently screened the titles and abstracts to identify relevant studies of clinical outcomes and complications after the intraoperative application of angiogenetic growth factors for the treatment of long bone fractures. If there was a disagreement between them, the final decision was made by the senior author (P.V.G.).

### 2.2. Inclusion Criteria and Study Selection

Studies that analysed the clinical outcome in patients after the intraoperative application of growth factors and cellular therapy for the treatment of long bone fractures and non-unions were identified. Only full-text articles were eligible for inclusion. Additional inclusion criteria included: (a) studies written in English, (b) studies concerning the application of osteoinductive molecules in human subjects, and (c) data on the outcome clearly given to each patient.

Published studies written in a language other than English were excluded. Studies without obtainable data or insufficient details about the type of intervention and the clinical outcomes, case reports, reviews, letters to the editor, conference abstracts, technical notes, and expert opinions were excluded. Research based only on in vitro or in vivo animal model results was also excluded.

### 2.3. Data Extraction

Two reviewers (E.P. and P.V.G.) examined all identified surveys, extracting data using a predetermined form. All data of each study were assembled in a Microsoft Excel spreadsheet, classified by orthopaedic intervention and type of osteoinductive factor. Characteristics extracted from clinical studies included the first author, the publication year, study design (cohort or randomised control trial), enrolled sample number in both control and treatment groups, patient demographics, anatomical site of interest, type of fracture (closed or open), orthopaedic procedure, outcomes regarding the frequency of non-union development, type and rate of detected complications, type of growth factor and or cellular therapy used and length of follow-up period. As the included studies did not report in detail the mean time between the primary intervention and reoperation and since the primary goal of our study was to compare the prevalence of the reoperations between the groups, this factor was not analysed due to the lack of accurate data. Fracture healing was defined clinically as the absence of pain on loading and radiologically as the presence of bridging callus formation in three out of four cortices on plain X-rays. The presence of duplicate studies was examined using the Endnote software (Clarivate Analytics, Philadelphia, PA, USA). All data were collected, summarised and analysed by two independent authors, A.K. and A.C.H.

### 2.4. Quality Assessment

The methodology of each study was assessed independently by the two senior authors (E.P. and P.V.G.) using the Newcastle-Ottawa quality assessment scale [19]. Included studies were graded in a three-category scale. Studies displaying a total score of 0–3, 4–6 and 7–9 were classified as poor, fair or good quality, respectively. A modified Jadad scale for clinical trials [20] was also used to evaluate the quality of included trials. A Jadad score greater than 4 was considered to be of high quality.

### 2.5. Statistical Analysis

Statistical analysis was performed using the Review Manager (RevMan) Version 5.4 (Nordic Cochrane Centre, Cochrane Collaboration, Copenhagen, Sweden). Moreover, MedCalc Meta-analysis Statistical software, v. 17.2 (MedCalc Software’s, Ostend, Belgium) was used to produce Egger’s test in order to test funnel plot asymmetry. The incidence of long-bone healing after the application of osteoinductive factors as well as the odd ratios (ORs) and the associated 95% Confidence Intervals (95% CI) were calculated. Heterogeneity between the trials was calculated by using Cochrane Q and the inconsistency (I^2^)–test. Values greater than 25%, 50% and 75% were considered as low, moderate and significantly heterogeneous, respectively. Therefore, a random effect model was used to calculate pooled ORs in the case of moderate and significant heterogeneity and the fixed effect model was used in the studies found with low heterogeneity. This was undertaken because, in the sensitivity analysis, the presentation of both models provides comprehensive evaluation of how differences in datasets affected the observed outcomes. Egger’s test and Forest plots were used to examine the risk of publication bias. The level of statistical significance was set at *p* ≤ 0.05.

## 3. Results

### 3.1. Search Results

**Figure 1:**
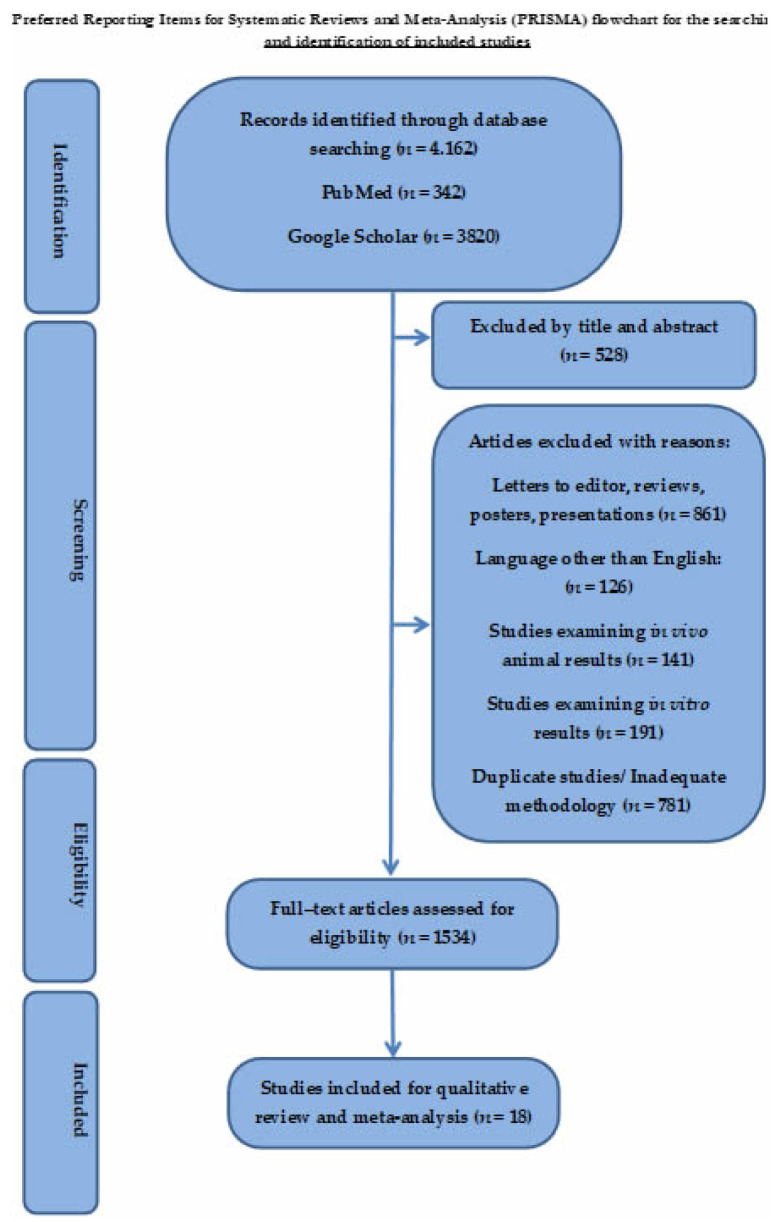
Preferred Reporting Items for Systematic Reviews and Meta-Analysis (PRISMA) flowchart for seeking and identifying included studies.

The literature search and cross-referencing resulted in 4162 references. After the initial evaluation of the studies based on the abstract and title, 1534 publications were included. Further analysis of the remaining papers resulted in the exclusion of 191 studies, as they followed an in vitro methodology only. In addition, 141 studies were excluded because they referred to animal models. Based on the inclusion criteria, 528 studies were excluded after reading the full article, while 861 and 126 studies were excluded because they were review articles or were written in languages other than English, respectively (Figure 1). Finally, 18 studies [21,22,23,24,25,26,27,28,29,30,31,32,33,34,35,36,37,38] published between 1996 and 2020 met our inclusion criteria. The data from each study are summarised in Table 1 and Table 2. Specifically, the studies included for meta-analysis are displayed in Table 1, while the studies selected for qualitative evaluation are shown in Table 2. The degree of agreement among the reviewers who examined the scientific quality of the included studies was strong, as shown in Table 3 and Table 4.

**Figure 2:**
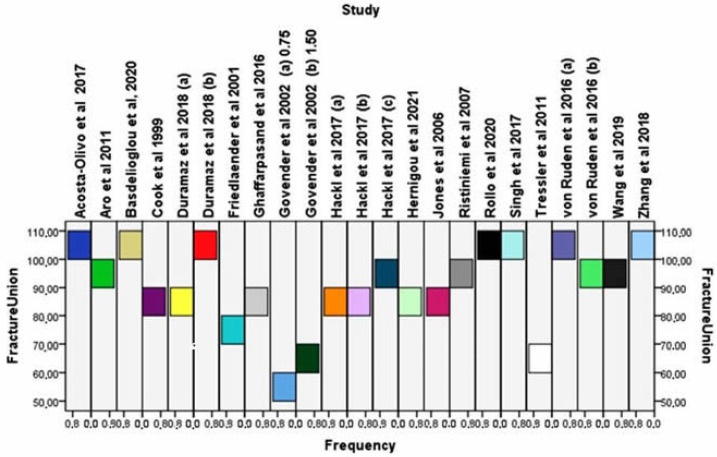
Prevalence of long bone fracture unions after the application of osteoinductive factors extracted from published bibliography [21,22,23,24,25,26,27,28,29,30,31,32,33,34,35,36,37,38].

According to the Newcastle-Ottawa scale and the modified Jadad score, all included trials were considered of high quality and were therefore deemed to be at a low risk of bias (Table 3 and Table 4). Moreover, after the evaluation of the funnel plot, all studies were found to lie within a 95% CI as represented by the inverted funnel, suggesting the absence of publication bias (Figure 2).

### 3.2. Clinical Application of Osteoinductive Factors

Overall, eighteen studies (Table 1) [21,22,23,24,25,26,27,28,29,30,31,32,33,34,35,36,37,38] analysed the potential healing enhancement after intraoperative application of osteoinductive factors, such as BMPs, and PRP and osteogenetic factors (MSCs) in long bone fractures. Four studies [26,29,30,36] reported results from different anatomic locations [26,29] or osteoinductive factors [30] or doses of the applied factor [36]. For optimal analysis, the above studies of Duramaz et al. [26], Von Ruden et al. [30], Govender et al. [36] and Hack et al. [29] were further divided in two [26,30,36] and three sub-categories, respectively and examined separately (Figure 2). All studies combined a broad spectrum of therapeutic techniques for fracture stabilisation such as intramedullary nailing (IMN), Ilizarov external fixation and open reduction and internal fixation (ORIF) and further osteoinductive and osteoconductive interventions such as autologous bone grafting. Furthermore, the efficacy of the applied factors in the enhancement of fracture healing was examined in close [10,24,34,37] and open fractures [21,22,27,32,34,36,37] as well as in non-union treatment [22,23,24,25,26,27,28,29,30,31,33,35,37,38].

Three studies analysed the efficacy of MSCs for sufficient osseous healing either in open tibial injuries associated with increased size of bone defects [21], in severe recalcitrant non-unions and pseudoarthrosis [24] as well as in previous infected tibial non-union fractures [25].

Ten studies [27,29,30,32,33,34,35,36,37,38] focused on the use of BMPs for enhancement of bone healing. Six of them [27,29,30,34,37,38] analysed the effectiveness of intra-operational application of BMP-7 in non-unions and in fractures with critical size defects of long bones of upper and lower limbs, while the impact of BMP-2 application was examined in five studies [30,32,33,35,36]. Furthermore, one clinical study compared the efficacy in upper and lower limb non-unions between BMP-2 and BMP-7 [30].

Similarly, five studies [22,23,26,28,31] assessed the effectiveness of PRP in accelerating the process of osseous healing. One study evaluated the activity of PRP in pseudoarthrosis healing [22]. A study compared the effectiveness of PRP and Hyperbaric Oxygen Therapy (HOT) for the treatment of aseptic tibial non-union using Ilizarov external fixation [23]. Due to the fact that a Cochrane systematic review did not reveal any clinical evidence supporting the efficacy of HOT application on the treatment of long bone fractures and non-unions [41], the HOT group was used as a control group in our analysis. Moreover, the correlation between fixation technique and PRP application was evaluated in three more studies [26,28,31]. One of them assessed the therapeutic potential of PRP in long bone oligotrophic non-unions treated using intramedullary nailing and whether exchange of implants was not reasonable. Likewise, the healing effect of PRP was interpreted in two further studies. The first analysed the application of PRP in delayed union of diaphyseal humeral fractures [28], while the second evaluated the role of PRP in long bone non-union fractures using IMN or ORIF fixation and autologous bone grafting.

Despite the fact that the efficacy of osteoinductive and cellular treatment in open fractures was described in seven studies [21,22,27,32,34,36,37], only five [21,27,32,35,36] met the predefined criteria, and were included for meta-analysis. Similarly, their effectiveness in closed fractures was reported by four researchers [22,23,34,37], but only two provided sufficient data and were included for meta-analysis (Table 1). It must be noted that the analysis for closed fractures was based only on data reported for the application of PRP on long bone fractures (Table 1). Specifically, the results of the application of BMP-2, BMP-7, PRP and MCS were analysed in two [32,36], three [27,34,37], one [21] and another one [22] studies, respectively. Similarly, the enhancement of bone healing in closed fractures by BMP-7 and PRP was examined in two studies each [35,37] and [22,23], respectively (Table 1). The safety and effectiveness of BMP-7 in non-union treatment was investigated in five studies [27,29,30,37,40], while the potency of BMP-2, PRP and MCSs was examined in three [30,33,34], five [22,23,26,28,31] and two studies [24,26], respectively (Table 1).

### 3.3. Statistical Results

#### 3.3.1. Overall Effectiveness

The frequency of fracture healing after the application of osteoinductive factors in each of the included studies is shown in Figure 2.

**Figure 3:**
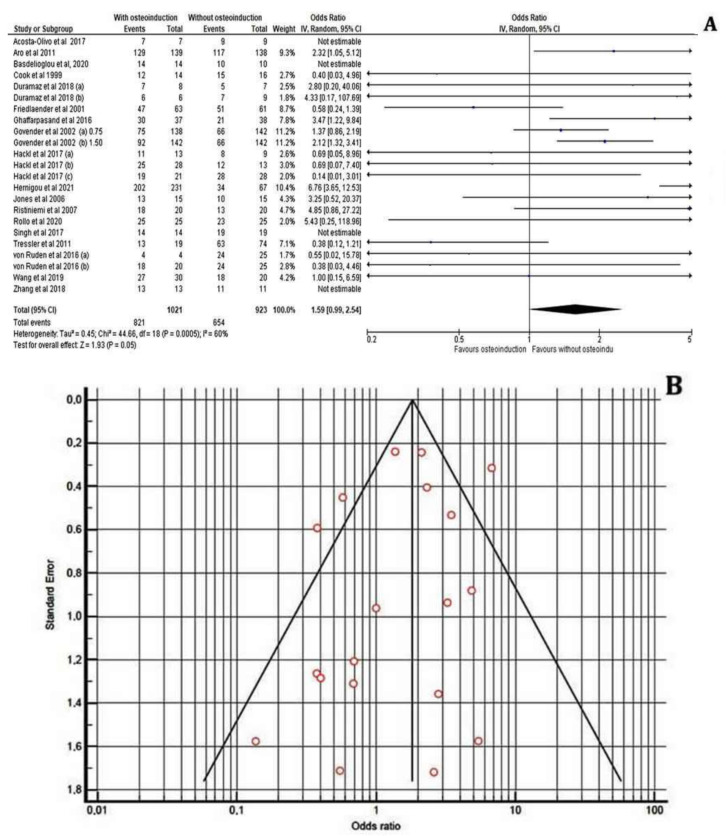
(**A**) Forest plots showing the overall likelihood of sufficient bone healing after the application or not of osteoinductive factors in fracture site. (**B**) Funnel plot of the Egger’s test utilised to evaluate the publication bias [21,22,23,24,25,26,27,28,29,30,31,32,33,34,35,36,37,38].

The overall effectiveness was found significantly increased in patients who received them compared to controls (I^2^ = 60%, 95% CI = 1.59 [0.99–2.54], Z =1.93, *p* = 0.05) (Figure 3).

#### 3.3.2. Subgroup Effectiveness Analysis (MCSs, PRP, BMP-7, BMP-2)

**Figure 4:**
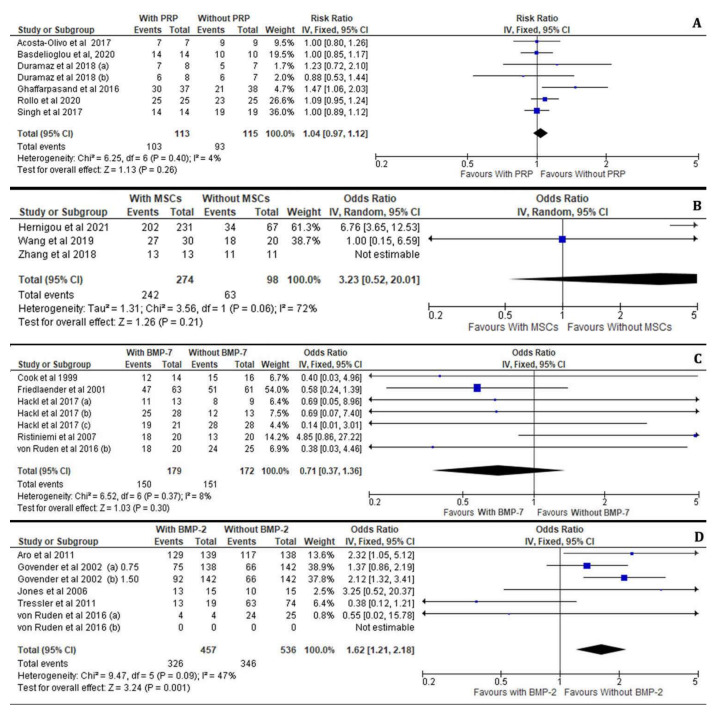
Forest plots displaying the effectiveness of PRPs (**A**), MSCs (**B**), BMP-2 (**C**) and BMP-7 (**D**) on the healing of long bone fractures in the treated group versus the non-treated (control) groups [21,22,23,24,25,27,28,29,30,31,32,33,34,35,36,37,38].

Regarding the healing rate at the fracture site, the patients who received PRP treatment for delayed union of diaphyseal humeral fractures as well as for pseudoarthrosis and oligotrophic non-unions of long bone fractures displayed no significant difference compared to the control groups (I^2^ = 4%, 95% CI = 1.04 [0.97–1.12], Z = 1.13, *p* = 0.26). Moreover, the potential for bone healing by PRP administration was not affected by the preferred surgical technique as these patients were treated either with ORIF, IMN or an Ilizarov external fixator with or without the addition of autologous bone grafts (Figure 4A).

Similarly, we have observed that the application of MSCs or BMP-7 was not statistically associated with an improved incidence of efficient healing outcome between the treated and non-treated cases with long bone fractures (I^2^ = 72%, 95% CI = 3.23 [0.52–20.01], Z = 1.26, *p* = 0.21) and (I^2^ = 8%, 95% CI = 0.71 [0.37–1.36], Z = 1.03, *p* = 0.30), respectively. The enrichment of autologous MSC at the fracture site was not associated with an advanced healing process in non-united open tibial fractures (Gustilo–Anderson Type II or III) which were treated with an Ilizarov technique or long bone non-unions treated by IMN (Figure 4B). Likewise, the application of BMP-7 in aseptic diaphyseal ulnar and/or radial shaft non-unions treated with compression plating and autologous bone grafting as well as in tibial non-unios after IMN was not found therapeutic (Figure 4C).

On the contrary, our meta-analysis showed that the application of BMP-2 (I^2^ = 47%, 95% CI = 1.62 [1.21–2.18], Z = 3.24, *p* = 0.001) in patients with open tibial shaft fractures treated with reamed and unreamed IMN or an external fixator was correlated with significantly increased rates of sufficient bone healing compared to the control patients. Overall, the addition of BMP-2 revealed a great enhancement of the healing process when surgical options such as ORIF, IMN or external fixation were employed in femur, tibia and humerus non-united open or closed fractures (Figure 4D).

**Figure 5:**
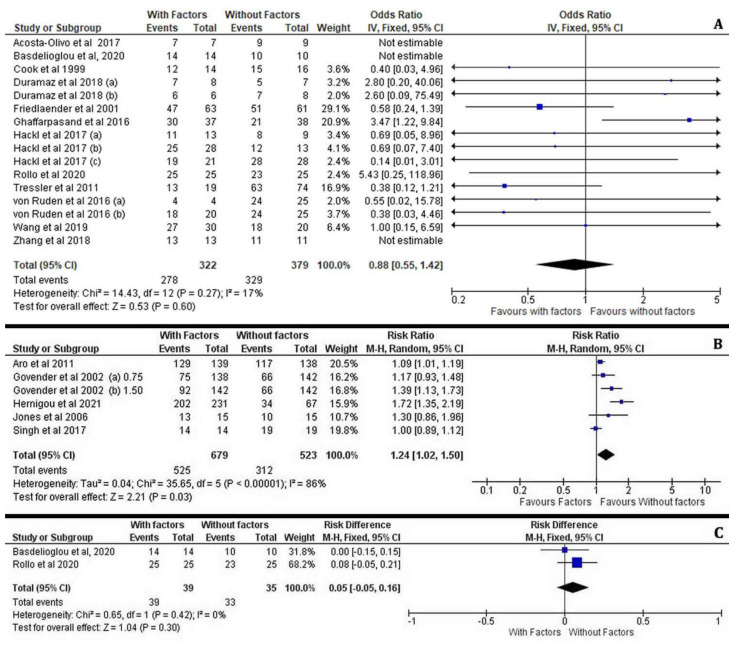
Forest plots showing the effectiveness of osteoinductive factors (**A**) on non-unions, (**B**) open and (**C**) close fractures compared to the control group of patients who have not received additional factors at the fracture site [21,22,23,24,25,26,27,28,29,30,31,32,33,35,36,37,38].

**Figure 6:**
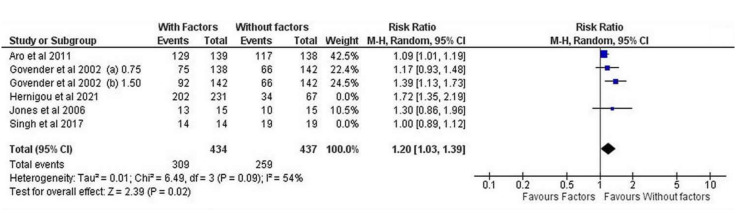
Forest plot displaying the efficacy of BMP-2 administration on open fractures of long bone diaphysis [21,27,32,35,36].

It is worth mentioning that interventions at the fractured non-united site require the most appropriate and individualised preoperative planning, which combines the best surgical fixation technique with the application of the most suitable bone stimulating factor. Overall, the present meta-analysis demonstrates different effectiveness on the healing potential when growth factors such as BMPs, PRPs and osteogenic factors (MSCs) are applied on long bone non-unions or open fractures. For instance, the post-surgical outcomes after the application of growth factors in patients suffering from humeral, radial, ulnar, femoral and tibial non-unions were not found significantly improved compared to those whose surgical treatment did not include the above growth factors (I^2^ = 17%, 95% CI = 0.88 [0.55–1.42], Z = 0.53, *p* = 0.60) (Figure 5A). However, the administration of bone stimulating factors such as BMP, MSC and PRP along with surgical treatment of long bone open fractures resulted in significantly enhanced healing outcomes compared to the avoidance of the above factors in the same type of fractures (I^2^ = 86%, 95% CI = 1.24 [1.02–1.50], Z = 2.21, *p* = 0.03) (Figure 5B), whereas in closed fractures the statistical difference was insignificant (I^2^ = 0%, 95% CI = 0.05 [−0.05–0.16], Z = 1.04, *p* = 0.30). It must be highlighted that application of BMP-2 for open fracture treatment was correlated with increased rates of bone healing (I^2^ = 54%, 95% CI = 1.20 [1.03–1.39], Z = 2.39, *p* = 0.02) (Figure 6).

#### 3.3.3. Persistent Non-Unions and Re-Fractures after the Application of Osteoinductive Growth Factors

**Figure 7:**
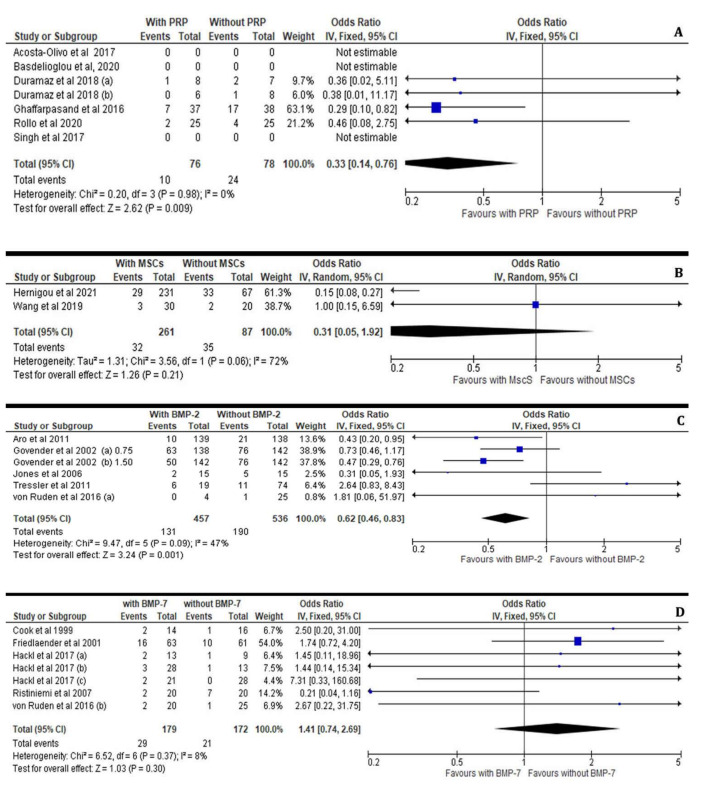
Forest plots demonstrating the incidence of refractures and non-unions despite the application of the osteoinductive factors PRPs (**A**), MSCs (**B**), BMP-2 (**C**) and BMP-7 (**D**) in the treated versus the non-treated (control) group [21,22,23,24,26,27,28,29,30,31,32,33,34,35,37].

The present meta-analysis further assessed the rates of persistent long bone non-unions and re-fractures at non sufficiently healed sites after the administration or not of growth factors, such as BMP, PRP and osteogenetic factor (MSC) in patients suffering from long bone non-unions. Hence, PRP-treated patients suffering non-united long bone fractures were not experiencing higher rates of persistent non-unions or re-fractures compared to their counterparts (I^2^ = 0%, 95% CI = 0.33 [0.14– 0.76], Z = 2.62, *p* = 0.009), as shown in Figure 7A. On the contrary, the application of autologous MSCs in open tibial fractures (with gaps less than 10 mm) Gustilo–Anderson Type II or III as well as in closed non-united long bone fractures was correlated with a mild increase (I^2^ = 72%, 95% CI = 0.31 [0.05–1.92], Z = 1.26, *p* = 0.21) of the risk for persistent non-unions and re-fractures, as shown in Figure 7B.

We have further considered the correlation between BMP application and persistent non-unions and re-fracture rates in patients suffering from non-unions after ORIF, IMN and external fixation treatment of closed and open long bone fractures. According to our meta-analysis, the administration of BMP-2 significantly (I^2^ = 47%, 95% CI = 0.62, Z = 3.24, *p*= 0.001) prevents persistent non-unions and re-fractures in treated patients compared to non-treated ones (Figure 7C). However, the application of BMP-7 at the fracture site in patients with long bone non-unions did not reveal any statistical difference (I^2^ = 8%, 95% CI = 1.41 [0.74–2.69], Z = 1.03, *p*= 0.30) in the healing rate of persistent non-unions and re-fractures compared to their non-treated counterparts, as shown in Figure 7D.

**Figure 8:**
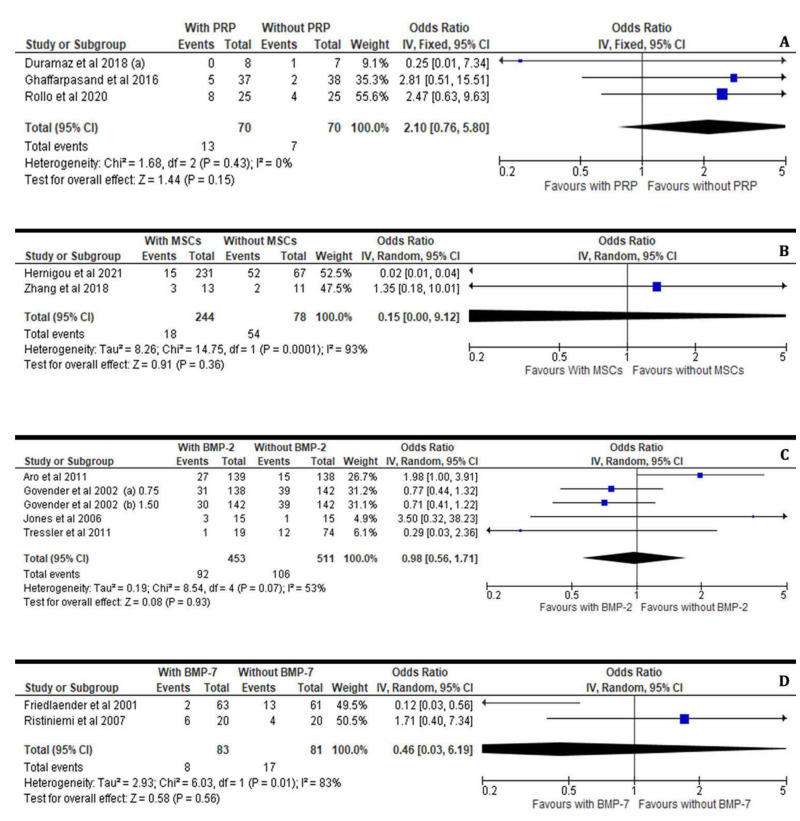
Forest plots showing the incidence of infections after the application of osteoinductive factors PRPs (**A**), MSCs (**B**), BMP-2 (**C**) and BMP-7 (**D**) in the treated versus the non-treated (control) groups [21,23,25,26,31,32,33,34,35,36,37].

In addition, the incidence of infections after application of bone stimulating factors was not significantly increased in patients who received PRP (I^2^ = 0%, 95% CI = 2.10 [0.76–5.80], Z = 1.44, *p* = 0.15), MSCs (I2 = 93%, 95% CI = 0.15 [0.00–9.12], Z = 0.91, *p* = 0.36), BMP-2 (I^2^ = 53%, 95% CI = 0.98 [0.56–1.71], Z = 0.08, *p* = 0.93) or BMP-7 (I^2^ = 83%, 95% CI = 0.46 [0.03–6.19], Z = 0.58, *p*= 0.56) for induction of the bone healing process compared with those who did not (Figure 8A–D).

**Figure 9:**
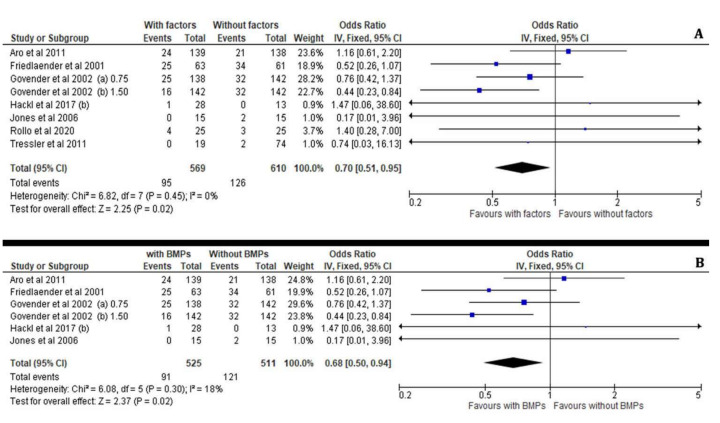
Forest plots showing the overall incidence of hardware failure after the application of osteoinductive factors (**A**) and after the use of BMPs only (**B**) compared with the non-treated (control) groups [23,29,32,33,35,36,37].

Likewise, the meta-analysis revealed that the risk for post operative hardware failure in patients receiving implant engraftment for their long bone non-union was overall significantly decreased after the application of osteoinductive and cellular factors (I^2^ = 0%, 95% CI = 0.70 [0.51–0.95], Z = 2.25, *p* = 0.02) as well as with the use of BMPs (I^2^ = 18%, 95% CI = 0.68 [0.50–0.94], Z = 2.37, *p* = 0.02) (Figure 9A,B).

#### 3.3.4. Osteoinductive Factors, Fixation Technique and Fracture Location

**Figure 10:**
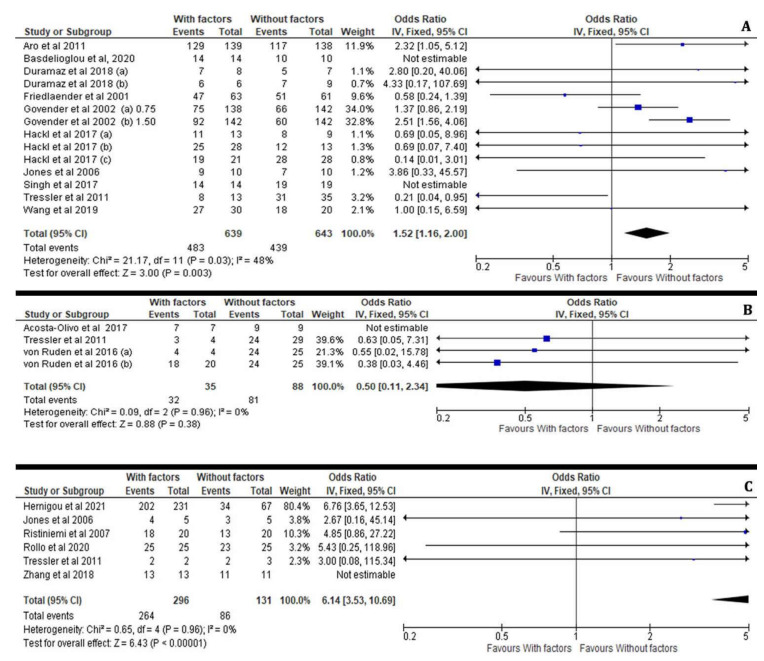
Forest plots presenting the effectiveness of osteoinductive factors in the bone healing process for treated versus non-treated patients in association with the preferred fixation technique for the fracture site: Intramedullary nailing (**A**), Open reduction and internal fixation (**B**), Ilizarov-external fixation technique (**C**) [21,22,23,24,25,26,27,28,29,30,32,33,34,35,36,37].

The application of the appropriate surgical technique for the management of long bone non-unions remains controversial in most cases and depends on the surgeon’s expertise and the personalised preoperative plan. The application of osteoinductive factors was significantly more effective in bone healing after IMN (I^2^ = 48%, 95% CI = 1.52 [1.16–2.00], Z = 3.00, *p* = 0.003) and Ilizarov external fixation (I^2^ = 0%, 95% CI = 6.14 [3.53–10.69], Z = 6.43, *p* < 0.00001) as opposed to the ORIF (I^2^ = 0%, 95% CI = 0.50 [0.11–2.34], Z = 0.88, *p* = 0.45) technique compared to the control groups (Figure 10A–C).

**Figure 11:**
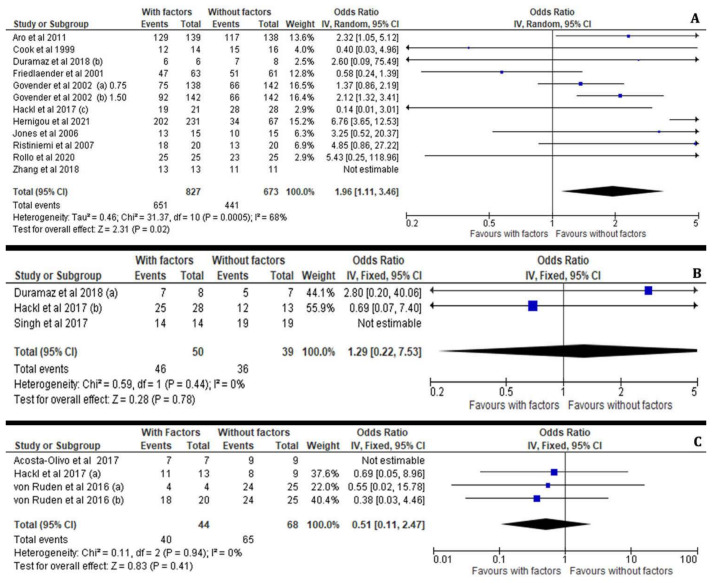
Forest plots presenting the effectiveness of osteoinductive factors in the bone healing process according to fracture anatomical location such as tibia (**A**), femur (**B**) and upper limb (**C**) [21,23,25,26,27,28,29,30,32,34,35,36,37,38].

Interestingly, our meta-analysis revealed that the major effectiveness of osteoinductive factors such as BMP, PRP and osteogenic factors (MSC) in open and closed injuries depends on the anatomical site of the fracture. Growth factors play the most significant key role in preventing non-unions when applied in tibial fractures (I^2^ = 68%, 95% CI = 1.96 [1.1–3.46], Z = 2.31, *p* = 0.02) compared to delayed non-unions in other anatomical sites, such as the femur (I^2^ = 0%, 95% CI = 1.29 [0.22–7.53], Z = 0.28, *p* = 0.78) and upper extremity (I^2^ = 0%, 95% CI = 0.51 [0.11–2.47], Z = 0.83, *p* = 0.41), respectively (Figure 11A–C).

## 4. Discussion

It is well established that efficient bone healing develops through fundamental overlapping physiologic stages: haematoma formation, inflammatory reaction, chondrogenesis and angiogenesis, osteogenesis and remodelling [39,40,41,42,43]. Therefore, the combination of osteogenesis and angiogenesis is essential for bone regeneration—reported as the “coupling process” [43]. The present study compared the growth factors currently available in clinical practice and exhibiting a critical role in the induction of “osteogenesis and angiogenesis coupling”. Although certain systematic reviews have already studied the involvement of BMPs, MSC and PRP in upper and lower extremity long bone fractures [44,45,46,47], the present meta-analysis compares all recent clinical available data on the osteoinductive potential of growth factors and cellular therapies, providing additional information about: (a) the distinct clinical effects of BMP-2 and BMP-7 application on bone healing, (b) the efficacy and safety of BMPs, MCSs and PRP in open and closed fractures or (c) in different anatomical locations of the upper and lower extremities and (d) after the application of different fixation techniques, revealing their optimal use in each category. The present meta-analysis demonstrated that patients diagnosed with open long bone fractures and complications of non-union or pseudarthrosis benefitted from implantation of osteoinductive growth factors.

BMPs are multifunctional cytokines belonging to the transforming growth factor-β (TGF-β) superfamily and their involvement in the osteogenesis process is well described [48,49,50,51]. In vitro and in vivo findings reported that BMP-2 promoted the angiogenetic effects in endothelial cells and induced the activation of circulating endothelial progenitor cells that possessed osteogenic and angiogenetic actions [49]. The present meta-analysis showed that the application of BMP-2 and BMP-7 at the fracture site was correlated with reduced fracture healing time, increased consolidation rates, and it was not accompanied by significant joint restriction or pain during motion. More specifically, according to a study by Govender et al., in patients with open tibial fractures administration of BMP-2 was accompanied by significant decrease of surgical failure risk, elimination of invasive interventions, such as bone grafting, a decrease in infections and secondary interventions rates compared to the control group [36,52]. This was also supported not only by the results of clinical cohorts (Table 2), but also by recent research studies [53,54]. Indeed, Kostiv et al., using immunohistological methods, reported that fracture non-union was the result of overproduction of cytotoxic and proapoptotic factors in chronic inflammation and dysfunction of BMP-2 expression [53]. Additionally, the application of BMP-2 composites materials during fixation of displaced femoral neck fractures with cannulated screws provided fewer complications, such as avascular necrosis and nonunion [49]. Concurring with the literature [51,52,53,54,55,56,57,58,59], our meta-analysis also confirmed the results of previous studies (Table 2) which supported the osteoinductive features of BMPs. However, our analysis showed that the increased healing rate, reduced repair time and post-operative infection rates were more prominent after the application of rhBMP-2 compared to rh-BMP-7 (Table 2). It is worth mentioning that consolidation rates after administration of rhBMP-2 appeared statistically higher compared to rhBMP-7 in two studies, the first by Haubruck et al. [50] and the second by Conway et al. [51]. Similarly, the systematic review of Sandler et al. confirmed that the application of BMP-2 in the treatment of upper extremity non-unions cases led to union in 117 days while in those treated with BMP-7 radiographic union was observed after 196 days [45].

Although adverse effects of BMP use were observed, such as heterotopic bone formation [55], synostosis and post-traumatic osteoarthritis [56], our meta-analysis showed that complication rates were low. This was in agreement with the results of Boraiah et al., who detected a limited number of heterotopic ossifications after rhBMP-2 application [52]. Additionally, no allergic or immunological reactions, severe deep infections, malignant transformation and persistent nerve palsy were observed. Our results were also consistent with the findings of several studies that reported limited side effects after the application of BMPs for the treatment of long bone fractures (Table 2). Indeed, the cohort studies of Kanakaris et al. [58,59], Giannoudis et al. [60] and Dimitriou et al. [61] that applied the combination of rhBMPs with autologous bone graft in a large sample for the treatment of persistent pseudarthrosis did not detect any infectious or allergic reactions.

Similarly, the risk for post operative hardware failure in patients receiving implant engraftment for their long bone fractures was overall significantly decreased after the application of BMPs. The above result was also supported by a study by Fuchs et al., which reported implant revision in only 3 out of 72 patients after the application of rhBMP-2 for the treatment of long bone non-union [62]. This could be explained by the fact that long bone fractures treated without BMP application were more prone to hardware failure due to either delayed healing or lower bone healing rates in general compared to BMP groups. It is particularly evident that the biomechanical stability of the construct is negatively affected in non-unions and is a major concern for future broken implants [63].

According to Caterini et al. [56], the administration of BMPs increased healing in non-unions of long bones either in one or two stage procedures, independently from the fixation technique (ORIF, IMN, external fixation, lag screws). However, our meta-analysis revealed that applied growth factors display a higher healing potential in fracture non-unions where IMN and external fixation (Ilizarov technique) are preferred, compared to ORIF.

Mesenchymal stem cells actively participate in angiogenesis and osteogenesis coupling through direct differentiation, cell contact interaction with endothelial lineage, and via releasing pro-angiogenic factors [64]. Furthermore, human adipose derived stem cells have an increased capacity to proliferate and differentiate into osteoblastic cells without the presence of growth factors [64]. These findings were also associated with the absence of genetic alterations, providing a safe method for clinical application [65]. Many studies (Table 2) demonstrated that MSC application at the non-union site was associated with increased fracture consolidation rates and with remarkably reduced fracture union times and hospitalisation periods [21,24,25]. The above findings were also associated with a high quality of health index [65]. Although the international literature reports that MSCs suppressed lymphocyte reaction and induced immunosuppressive properties [61], our analysis demonstrated that adverse effects after MSC application were extremely uncommon [21,24,25]. Despite the fact that many studies reported increased healing outcomes after the administration of MSCs in patients with atrophic and hypertrophic non-unions, in critical-size bone defects as well as in depressed tibial plateau fractures [66,67,68,69,70], our meta-analysis did not reveal statistical differences in the healing process or in the prevention of refractures after MSC application compared to the control group. Contrariwise, our analysis confirmed the safety of MSCs application as their use was not associated with allergic reactions, deep infections, ectopic neo-formations and neoplastic transformations [71].

Similarly, the application of PRP has also demonstrated an important role during the fracture repair process, due to its ability to induce complex inflammatory responses at the bone defect site, not only by activating angiogenesis but also by providing growth factors, such as VEGF, PDGF, TGF-β and IGF [71]. Although our meta-analysis did not reveal significant differences in the healing rate of patients with long bone fractures that received PRP treatment compared to the control group, the examination of the included studies showed that PRP use was accompanied by increased success rates for non-union treatment, decreased fracture healing periods and very good overall functional outcomes. Our findings are in line with the results of a recent meta-analysis that detected higher healing rates and a shorter consolidation duration accompanied by significant pain relief after the treatment with PRP for long bone delayed union and nonunion [45]. This was also supported by the study of Bielecki et al., where PRP use resulted in complete healing in all patients with delayed union fractures [72]. Moreover, no complications such as damage to blood vessels, haematomas, delayed wound healing, permanent nerve damage and post-operative infections were observed. Studies (Table 2) also demonstrated that PRP is a useful growth factor in non-unions of long bones, boosting the healing process without reported complications, such as allergic reactions, deep infections, ectopic neo-formations, neoplastic transformations or re-fractures, in line with our meta-analysis [73,74,75,76]. However, Calori et al. reported a superior osteoinductive activity of BMP-2 compared with PRP; this was consistent with our results [77].

An interesting finding of our analysis was that the efficacy of growth factors was statistically correlated with the fracture anatomical location. Indeed, the bone healing process was more prominent in tibial than in femoral and upper limb fractures. Our results were in line with a recent study that reported increased achievement of healing after BMP-2 in tibial fractures compared to humeral fractures [62]. A possible explanation to this finding could be that the relationship between tibial bone structure and axial mechanical loading may provide an exceptional biomechanical environment for growth factor activity. Mechanical loading is a significant factor of bone remodelling and the absence of mechanical signals was accompanied by increased bone resorption and reduced bone formation [77,78,79,80]. Furthermore, mechanical strain increased the secretion of chemokines and the recruitment of MSCs to bone surface, promoting bone formation [81]. Recent findings have also detected links between induction of endosteal progenitors and mechanical loading-induced growth factors released from osteoblastic cells [80]. As tibial bones receive increased axial loading [81], we can assume that these benefit the most from application of osteogenic factors after fracture.

## 5. Study Limitations

The study limitations include a language bias, as only studies written in English were reviewed and thus some studies were not included in our analysis. Another limitation was that the examined studies contained mixed groups of open and closed fractures and information about the number and outcome of closed fractures was based on data concerning only PRP application. The use of a HOT group as control group in the included study by Rollo et al. [23] could be another possible limitation. Further limitations were the variability of treatment protocols, the different selection criteria or follow-up periods. Additional limitations to consider were the differences in methodological approaches between the studies, the conditions under which these were conducted, other confounding factors that were not taken into consideration or the increased risk of bias of the selected studies, especially of those that were not evaluated. Additionally, the assessment of callus formation was based on clinical signs and plain radiographs that could be associated with uncertain healing outcome as CT-scan is the gold standard for the diagnosis of the healing process [59]. We must also draw attention to the fact that studies including thoroughly negative assessments about intraoperative growth factor application may face difficulties in being published by peer-reviewed journals.

## 6. Conclusions

Despite the limitations of our study, our results tend to support the efficacy of osteoinductive factors, such as BMP-2, BMP-7, PRP and osteogenic factors (MSCs) in the long-bone healing process. The intraoperative application of these factors was considered safe and with an effective intervention that enhanced angiogenesis and was linked to augmented fracture healing, reduced repair time and very good functional scores. Therefore, our meta-analysis suggests that the combination of a precise fixation technique with application of osteoinductive/osteogenetic factors supplies an optimum environment to support the healing microenvironment at the fracture site, with a positive impact on clinical outcomes.

## Figures and Tables

**Table 1:** Clinical characteristics of the studies included for meta-analysis with osteoinductive factors for long bone fracture healing (N/A: applicable, Pts: patients, rh: recombinant human, MSCs: Mesenchymal cells, PRP: Platelet Rich Factor, BMP: Bone Morphogenetic Protein).

Author/Year/Country	Type of Study	Number of Open Fractures (*n*)	Number of Closed Fractures (*n*)	Number of Non-Unions (*n*)	Type of Osteosynthesis	Osteo-Inductive Factors Applied/Scaffolds	Patients Treated with Osteoinductive Growth Factors (*n*)	Patients Treated without Osteoinductive Growth Factors (*n*)	Median Follow-Up (Months)	Outcome
Hernigou et al. 2021, France [21]	Prospective randomised cohort study	Open fractures (with gap less than 10 mm) Gustilo - Anderson Type II or III *n* = 231 treated with Bone marrow concentrate *n* = 67 control (no early graft) patients *n* = 76 treated with an early, standard of care, iliac bone graft	No	No	External fixation/Ilizarov	Bone marrow with mesenchymal stem cells (MSCs)	*n* = 231 Bone marrow group (MSCs)	*n* = 67 (control group = no early graft) *n* = 76 (standard iliac bone graft)	09	Bone union in: 50.7% Control group 86.8% Iliac Bone graft group 87.4% Bone marrow group (MSCs)
Başdelioğlu et al. 2020, Turkey [22]	Retrospective study	*n* = 1 in PPR group *n* = 1 in the control group	*n* = 13 in PRP group *n* = 09 in the control group	*n* = 14 in the PRP group *n* = 10 in the control group	ORIF: *n* = 7 PRP *n* = 4 not PRP IMN: PRP *n* = 3 not PRP *n* = 1 ILIZAROV: *n* = 1 PRP *n* = 1 not PRP CAST: *n* = 1 PRP *n* = 2 not PRP BANDAGE: *n* = 1 PRP, *n* = 0 not PRP	Autologous platelet-rich plasma (PRP), autologous bone graft allograft	*n* = 14	*n* = 10	03	Fracture healing in both groups Statistically significant difference in time healing (5.3 months in PRP group and 11.3 in control group) No complications
Rollo et al. 2020, Italy [23]	Retrospective study	No	*n* = 50	*n* = 50 Type B according ASAMI tibial non union	External fixation/Ilizarov	PRP or Hyperbaric Oxygen Therapy (HOT)	*n* = 25 with PRP	*n* = 25 with HOT	12	Ilizarov technique plus PRP (or HOT) does not improve the functional outcomes but allows a more rapid healing of the regenerated bone
Wang et al. 2019, China [24]	Retrospective case control study	N/A	N/A	*n* = 50 Humerus: 5 Radius/ulna:3 Femur: 23 Tibia/fibula: 19	ORIF	Mesenchymal stem cells (MSC), β-tricalcium phosphate scaffolds, autologous bone graft	*n* = 30	*n* = 20	09	Healing of bone defects in 45 pts (success rate: 90%)
Zhang et al. 2018, China [25]	Prospective randomised cohort study	N/A	N/A	*n* = 24 Infected tibial non-union fractures Non-union gap between 3 and 12 cm	External fixation/Ilizarov	Autologous mesenchymal stem cells (MSC)	*n* = 13	*n* = 11	16	Significant decrease in union time and hospitalisation period in the MSC group
Duramaz et al. 2018, Turkey [26]	Retrospective study	N/A	N/A	*n* = 29 Long bone oligotrophic non-unions	Femur: *n* = 8 PRP, *n* = 7 IMN exchange Tibia: *n* = 6 PRP, *n* = 8 IMN exchange	PRP	*n* = 14	*n* = 15	09	Percutaneous PRP application significantly affected union rate, but without significant difference compared to exchange intramedullary nailing. PRP remains a minimal invasive technique instead of exchange intramedullary nailing.
Singh et al. 2018, UK [27]	Prospective randomised cohort study	*n* = 31	*n* = 11	*n* = 42 Non-union of the upper limb bones Radius and ulna (*n* = 25), humerus (*n* = 14), clavicle (*n* = 3)	*n* = 31 had operative interventions (ORIF) *n* = 11 non-operative	rhBMP-7 autologous bone graft	*n* = 14	*n* = 19	12–36	Healing of non-union in 40 pts, Partial union in 2 pts DASH score: 33.7 Heterotopic bone formation in 2 pts
Acosta-Olivo et al. 2017, Mexico [28]	Prospective randomised cohort study	N/A	N/A	*n* = 16 Delayed union of diaphyseal humeral fractures	Locking compression plate (LCP) fixation	iliac crest autograft using platelet-rich plasma (PRP)	*n* = 08	*n* = 08	09	PRP promotes earlier bone consolidation (19.9 weeks compared to 25.4 weeks in control group)
Hackl et al. 2017, Germany [29]	Retrospective comparative study	N/A	N/A	*n* = 112 Treatment of aseptic diaphyseal Non-union humerus: 19 femur: 37 tibia: 47	Locking compression plate (LCP) fixation	Recombinant human Bone Morphogenetic Protein-7 (rhBMP-7)	*n* = 62 with rhBMP-7	*n* = 50	12	Aseptic diaphyseal non-union in humerus, femur, and tibia healed irrespectively of additional rhBMP-7 application.
Von Ruden et al. 2016, Germany [30]	Prospective randomised cohort study	N/A	N/A	*n* = 49 Aseptic diaphyseal ulnar and/or radial shaft non-union	Compression plating (ORIF)	With or without human recombinant Bone Morphogenetic Proteins BMP-2 and BMP-7	BMP-2 (*n* = 4) BMP-7 (*n* = 20)	*n* = 25	06–54 (Median 15)	Atrophic/oligotrophic forearm non-union healed irrespective of additional application of BMP combined with autologous bone grafting.
Ghaffarpasand et al. 2016, Iran [31]	Prospective randomised double-blind placebo controlled trial	N/A	N/A	Long bone non-union fracture Hypertrophic PRP: *n* = 23 Placebo: *n* = 25 Oligotrophic PRP: *n* = 9 Placebo: *n* = 11 Atrophic PRP: *n* = 5 Placebo: *n* = 2 Femur PRP: *n* = 16 Placebo: *n* = 19 Tibia PRP: *n* = 14 Placebo: *n* = 12 Humerus PRP: *n* = 6 Placebo: *n* = 5 Ulna PRP: *n* = 1 Placebo: *n* = 2	IMN or ORIF along with autologous bone graft.	PRP	*n* = 37 5 mL PRP	*n* = 38 5 mL normal saline (placebo)	09	Healing rate significantly higher in the PRP group compared to placebo (81.1% vs. 55.3%; *p* = 0.025). Limb shortening significantly higher in the placebo group (2.61 ± 1.5 vs. 1.88 ± 1.2 mm; *p* = 0.030). The PRP group had lower pain scores (*p* = 0.003) and shorter healing duration (*p* = 0.046).
Aro et al. 2011, Finland [32]	Prospective randomised cohort study	*n* = 277 Open tibial fractures Gustilo–Anderson Type IIIB	No	No	Reamed IMN	rhBMP-2, bovine derived collagen type-I	*n* = 139	*n* = 138	Healed fracture at 13 and 20 weeks	Healing was not accelerated in the BMP-2 group Deep infections were more common in the BMP-2 group
Tressler et al. 2011, USA [33]	Retrospective study	N/A	N/A	*n* = 93 Long bone fractures: femur, tibia, and humerus	External fixation/Ilizarov: rhBMP-2: *n* = 2 autograft: *n* = 3 ORIF: rhBMP-2: *n* = 4 autograft: *n* = 29 IMN: rhBMP-2: *n* = 13 autograft: *n* = 35 Nonoperative: rhBMP-2: *n* = 0 autograft: *n* = 7	rhBMP-2 mixed with cancellous allograft vs compared iliac crest autograft	*n* = 19	*n* = 74	20.0 ± 17.7	No statistical difference in the rate of healing between treatment groups (rhBMP-2 = 68.4% vs Control = 85.1%, *p* = 0.09) rhBMP-2 may be a suitable alternative to autologous iliac bone graft, with shorter operative time and reduced intraoperative blood loss
Ristiniemi et al. 2007, Finland [34]	Prospective randomised cohort study	*n* = 04 Distal tibial fractures	*n* = 36 Distal tibial fractures	All fractures united	External fixation/ Ilizarov	rhBMP-7, bovine collagen	*n* = 20	*n* = 20	12	Healing of fractures in all pts, Delayed healing in 2 pts Time healing and external fixation application of the BMP-7 group was significantly shorter
Jones et al. 2006, USA [35]	Prospective randomised cohort study	*n* = 27 (24: Gustilo–Anderson type-IIIA or IIIB) Diaphyseal tibial fracture with residual defect	*n* = 03 Diaphyseal tibial fracture with residual defect	No	IMN or External fixation/Ilizarov	1st Group (*n* = 15): autologous bone graft 2nd Group (*n* = 15): rhBMP-2 with cancellous bone chips allograft soaked on absorbable collagen sponge	*n* = 15	*n* = 15	12	Similar healing rates between the groups Deep infections in 4 pts (*n* = 1 of 1st group and *n* = 3 of the 2nd group), without immunological reactions
Govender et al. 2002, Multicentre study [36]	Prospective randomised cohort study	*n* = 450 Open tibial shaft fractures	No	No	Reamed and undreamed IMN	rhBMP-2, bovine derived collagen type-I	*n* = 300	*n* = 150	12	The rhBMP-2 group showed accelerated wound and fracture healing and reduction in frequency of secondary operations and infection rates
Friedlaender et al. 2001 USA [37]	Prospective randomised comparative cohort study	*n* = 115	*n* = 09	*n* = 124 Non-union of the tibia	IMN	rhBMP-7 (*n* = 124) bovine derived collagen type-I (*n* = 63) autologous bone graft (*n* = 61)	*n* = 63	*n* = 61	24	Healing of non-union in 104 pts, Consolidation rate similar between the groups, without deep infection or allergic reactions
Cook et al. 1999, USA [38]	Prospective randomised cohort study	N/A	N/A	*n* = 30 *n* = 31 Tibial non-union	Reamed IMN	BMP-7 or autologous iliac crest bone	*n* = 14 (15 non-union)	*n* = 16	09	Similar healing characteristics between BMP-7 application and autologous iliac crest bone. Advantages of BMP-7: - no donor site complications - less blood loss shorter operative time

**Table 2:** Clinical data of studies included for qualitative examination demonstrating significant results for long bone fracture healing treatment after application of osteoinductive growth factors (pts: patients, rh: recombinant human).

Author/Year/Country	Type of Study	Indications/Surgical Interventions	Osteoinductive Growth Factor Applied/Scaffolds	Patients Treated with Osteoinductive Growth Factors (*n*)	Median Follow-Up (Months)	Outcome
Haubruck et al. 2018, Germany [39]	Retrospective comparative cohort study	Non-union of the long bones of the lower limbs one (*n* = 58) or two stage (*n* = 98) procedures with plates (*n* = 85), IMN (*n* = 65), external fixation (*n* = 4), lag screws (*n* = 2)	rhBMP-2 (*n* = 46), rhBMP-7 (*n* = 110) autologous bone graft PMMA cement spacer with gentamycin	156 (F/M: 68/82)	12	Pts with rhBMP-2 showed a statistically higher consolidation rate
Caterini et al. 2016, Italy [40]	Prospective cohort study	Atrophic non-union of the humeral shaft/internal fixation with compression plate	rhBMP-7, autologous bone graft hydroxyapatite pellets	12 (F/M: 8/4)	7.3	Healing of non-union in all pts, without humeral clinical instability
Conway et al. 2014, USA [41]	Retrospective comparative cohort study	Non-union of the long bones (*n* = 214 limbs) Tibia (*n* = 78), femur (*n* = 66), humerus (*n* = 70)	rhBMP-2, rhBMP-7, autologous bone graft, allograft	175 (F/M: 81/94)	17	Healing was increased in the BMP-2 group (93%) Time healing was reduced in the BMP-2 group Complication rates were lower in the BMP-2 group
Starman et al. 2012, USA [42]	Retrospective cohort study	Acute (*n* = 35) and aseptic and septic non-union (*n* = 81) fractures of the femur (*n* = 62), tibia (*n* = 45), fibula (*n* = 2), clavicle (*n* = 1), humerus (*n* = 5), ulna (*n* = 1)	rhBMP-2, without graft (*n* = 31), autologous bone graft (*n* = 13), allograft (*n* = 67), allograft and autograft (*n* = 05)	116 (F/M:49/67)	11	Healing of non-union in 76 pts, revision surgery in 30 pts
Papanna et al. 2012 UK [43]	Retrospective cohort study	Persistent non-unions of the upper and lower limbs femur (*n* = 9), tibia (*n* = 21), foot and ankle (*n* = 5), clavicle (*n* = 3), humerus (*n* = 10), ulna and radius (*n* = 4)	rhBMP-7, bovine derived collagen type-I, tri-calcium phosphate crystals	52 (F/M:22/30)	13.9	Clinical and radiological union in 48 pts, Joint stiffness (*n* = 3) Synostosis (tibiofibular, *n* = 1) Post-traumatic OA (*n* = 1) Without deep infection or allergic reactions
Kanakaris et al. 2009, UK [44]	Prospective cohort study	Atrophic, aseptic non-union of the femur (22 closed, 08 open) /Intramedullary Nailing (*n* = 17), ORIF (*n* = 10), Ilizarov circular frame (*n* = 3)	rhBMP-7, autologous bone graft	30 (F/M:8/22)	30	Healing of non-union in 26 pts, Revision surgery in 04 pts, without deep infection or allergic reactions
Giannoudis et al. 2009, UK [45]	Retrospective cohort study	Atrophic, aseptic non-union of long bones (humeral:07, femoral:19, tibial:19, 31 closed 14 open) /Intramedullary Nailing, ORIF	rhBMP-7, autologous bone graft	45 (F/M:13/32)	24.8	Healing of non-union in all pts, Median pain VAS:9, without deep infection or allergic reactions
Kanakaris et al. 2008, UK [46]	Retrospective and Prospective cohort study	Atrophic, aseptic non-union of the tibia (39 closed- 29 open) Intramedullary Nailing (*n* = 26), ORIF (*n* = 33), External Fixation (*n* = 8), non-operatively (*n* = 1)	rhBMP-7, autologous bone graft	68 (F/M:18/50)	18	Healing of non-union in 61 pts, revision surgery in 07 pts, median health VAS: 8.2, without deep infection or allergic reactions
Dimitriou et al. 2005, UK [47]	Prospective randomised cohort study	Persistent non-unions of the upper and lower limbs Tibial (*n* = 10), femoral (*n* = 8), humeral (*n* = 3), ulnar (*n* = 3), patellar (*n* = 1), clavicular (*n* = 1) treated with IMN or ORIF	1st Group (*n* = 9): rhBMP-7 autologous bone graft injection of bone marrow 2nd Group (*n* = 15): rhBMP-7	25 (F/M: 06/19)	15.3	Healing of non-union in 24 pts, without deep infection or allergic reactions
Bhattacharjee et al. 2019, UK [48]	Prospective cohort study	Severe recalcitrant atrophic (*n* = 29) and hypertrophic (*n* = 06) Non-union of the tibia (*n* = 16) and femur (*n* = 19)	Mesenchymal stem cells (MSC), Hydroxyapatite, tricalcium phosphate, calcium phosphate, serum	35 (F/M: 14/21)	30	Healing of bone defects in 21 pts (success rate: 60%) Significant increase of quality of health index (Eq5D) Sepsis in *n* = 1 pt.
Dilogo et al. 2019 Indonesia [49]	Prospective experimental study	Critical size bony defects with previously failed surgical attempts	Mesenchymal stem cells (MSC), Hydroxyapatite, rhBMP-2	06 (F/M: 02/04)	19	Healing of bone defects in all pts
Chu et al. 2018, China [50]	Retrospective comparative cohort study	Depressed tibial plateau fractures	Mesenchymal stem cells (MSC), β-tricalcium phosphate scaffolds	39 (F/M: /24/15)	30.5	Healing of bone defects in all pts
Giannotti et al. 2013, Italy [51]	Prospective experimental study	Atrophic pseudarthrosis of the upper limb	Mesenchymal stem cells (MSC) embedded in fibrin clot, autologous bone graft, homologous bone chips, synthetic bone chips	08 (F/M: 4/4)	76	Healing of non-union in all pts One pt had a 2nd intervention Without allergic reactions, deep infections, ectopic neo-formations or neoplastic transformations Absence of re-fracture
Malhotra et al. 2015, India [52]	Prospective cohort study	Non-union of the long bones Tibia (*n* = 35), femur (*n* = 30), humerus (*n* = 11), radius and ulna (*n* = 18)	Autologous platelet-rich plasma (PRP)	94 (F/M: 28/66)	3	Healing of non-union in 82 pts
Golos et al. 2014, Poland [53]	Retrospective cohort study	Delayed union of the long bones	Autologous platelet-rich plasma (PRP)	132 (F/M: 53/79)	-	Healing of non-union in 108 pts
Galasso et al. 2008, Italy [54]	Prospective cohort study	Atrophic aseptic diaphyseal non-unions of long bones (humeral:03, femoral:08, tibial:11), Expandable Intramedullary Nailing	Autologous platelet-rich plasma (PRP)	22 (F/M:09/13)	13	Healing of non-union in all pts Mean time to union: 21.5 weeks One pt suffered moderate pain and limitation of the abduction Without complications like haematomas, infections, delayed wound healing

**Table 3:** Study quality of the included studies based on the Newcastle–Ottawa scale (* Follow-up more than 24 months; ** Lost to follow-up rate more than 10% is considered inadequate).

Author Year	Representativeness of the Exposed Cohort	Selection of the Nonexposed Cohort	Ascertainment of Exposure	Demonstration That Outcome of Interest Was Not Present at Start of the Study	Comparability of Cohorts on the Basis of the Design or Analysis	Assessment of the Outcome	Follow up Long Enough for Outcomes *	Adequacy of Follow-Up of Cohort **	Total	Quality
Hernigou et al. 2021, France [21]	1	1	1	1	2	1	1	1	09	Good
Basdelioglu et al. 2020, Turkey [22]	1	1	1	1	2	1	0	0	08	Good
Rollo et al. 2020, Italy [23]	1	1	1	1	2	1	0	0	08	Good
Wang et al. 2019, China [24]	1	1	1	1	2	1	1	1	09	Good
Zhang et al. 2018, China [25]	1	1	1	1	2	1	1	1	09	Good
Duramaz et al. 2018, Turkey [26]	1	1	1	1	2	1	0	1	08	Good
Singh et al. 2018, UK [27]	1	1	1	1	2	1	0	0	07	Good
Acosta-Olivo et al. 2017, Mexico [28]	1	1	1	1	2	1	0	0	07	Good
Hackl et al. 2017, Germany [29]	1	1	1	1	2	1	1	1	09	Good
Von Ruden et al. 2016, Germany [30]	1	1	1	1	2	1	1	1	09	Good
Ghaffarpasand et al. 2016, Iran [31]	1	1	1	1	2	1	1	1	09	Good
Aro et al. 2011, Finland [32]	1	1	1	1	2	1	0	1	08	Good
Tressler et al. 2011, USA [33]	1	1	1	1	2	1	1	1	09	Good
Ristiniemi et al. 2007, Finland [34]	1	1	1	1	2	1	0	1	08	Good
Jones et al. 2006, USA [35]	1	1	1	1	2	1	0	0	07	Good
Govender et al. 2002, Multicentre study [36]	1	1	1	1	2	1	0	0	07	Good
Friedlaender et al. 2001, USA [37]	1	1	1	1	1	1	1	0	07	Good
Cook et al. 1999, USA [38]	1	1	1	1	1	1	1	0	07	Good

**Table 4:** Study quality of the included studies based on the modified Jadad scale (*: indicates one point, **: indicated two points).

Author(s) Year	Randomization	Concealment of Allocation	Double Blinding	Total Withdrawals and Dropouts	Total	Quality
Hernigou et al., 2021, France [21]	**	*	*	**	06	Good
Basdelioglu et al., 2020, Turkey [22]	*	*	*	*	04	Good
Rollo et al., 2020, Italy [23]	*	*	*	*	04	Good
Wang et al., 2019, China [24]	**	*	*	**	06	Good
Zhang et al., 2018, China [25]	**	*	*	*	05	Good
Duramaz et al., 2018, Turkey [26]	**	*	*	**	06	Good
Singh et al., 2018, UK [27]	*	*	*	*	04	Good
Acosta-Olivo et al., 2017, Mexico [28]	*	*	*	*	04	Good
Hackl et al., 2017, Germany [29]	**	*	*	**	06	Good
Von Ruden et al., 2016, Germany [30]	**	*	*	**	06	Good
Ghaffarpasand et al., 2016, Iran [31]	**	*	*	**	06	Good
Aro et al., 2011, Finland [32]	**	*	*	**	06	Good
Tressler et al., 2011, USA [33]	*	*	*	*	04	Good
Ristiniemi et al., 2007, Finland [34]	*	*	*	*	04	Good
Jones et al., 2006, USA [35]	**	*	*	**	06	Good
Govender et al., 2002, Multicentre study [36]	*	*	*	*	04	Good
Friedlaender et al., 2001, USA [37]	**	*	*	*	05	Good
Cook et al., 1999, USA [38]	*	*	*	*	04	Good

## Abbreviations

PRISMA: Preferred Reporting Items for Systematic Reviews and Meta-Analyses; BMPs: Bone Morphogenetic Proteins; MSCs: Mesenchymal stem cells; PRP: Platelet Rich Plasma; IMN: intramedullary nailing; ORIF: open reduction and internal fixation; pts: patients; rh: recombinant human.
